# Nephroprotective Effects of L-Carnitine against Contrast-Induced Nephropathy in Patients Undergoing Percutaneous Coronary Intervention: A Randomized Open-Labeled Clinical Trial

**Published:** 2017-04

**Authors:** Mohammad Mohammadi, Azita Hajhossein Talasaz, Mohammad Alidoosti, Hamid Reza Pour Hosseini, Kheirollah Gholami, Arash Jalali, Hamid Aryannejad

**Affiliations:** 1 *Department of Clinical Pharmacy, Faculty of Pharmacy, Tehran University of Medical Sciences, Tehran, Iran.*; 2 *Tehran Heart Center, Tehran University of Medical Sciences, Tehran, Iran*

**Keywords:** *Coronary artery disease*, *Angioplasty*, *Contrast medium*, *Acute kidney injury*, *Kidney diseases*

## Abstract

**Background: **L-carnitine may prevent the incidence of AKI by its antioxidant effects and improving circulation in ischemic conditions. The goal of this trial was to evaluate the impact of L-carnitine on contrast-induced nephropathy in patients undergoing elective PCI.

**Methods:** Totally, the patients were randomly allocated to 2 groups. The treatment group received 1 g of L-carnitine orally 3 times a day, 24 hours before the procedure (3 g before PCI) and 2 g after PCI, whereas the control group did not receive L-carnitine. In both groups, the plasma level of neutrophil gelatinase-associated lipocalin (NGAL) was measured at baseline and 12 hours after PCI.

**Results:** Our study was conducted on 202 patients (including 91 vs. 111 patients in the treatment and the control group; 31 (34.1%) vs 33 (29.7%) female in carnitine and control group; and ages include 62.0 ± 9.0 vs 57.0 ± 11.2 years). The median plasma levels of NGAL were not different between the carnitine and control groups at baseline (57 [IQR: 22 – 255] vs. 54 [IQR: 29 – 324]; p value = 0.155) and 12 hours after PCI (71 [IQR: 52 – 129] vs. 70 [IQR: 46 – 153]; p value = 0.925), but the changes in the plasma NGAL from baseline to 12 hours after PCI were different between the 2 groups (5 [IQR:-147 – 30] vs. 17 [IQR: -21 – 41]; p value = 0.010).

**Conclusion:** Our results showed that oral L-carnitine was able to prevent an increase in NGAL following contrast medium administration in patients undergoing PCI. More studies should be performed to fully elucidate the nephroprotective effects of L-carnitine.

## Introduction

The radiological contrast medium is one of the most common causes of renal injury (11%–12% of all cases) and is a significant, albeit underestimated, problem in clinical practice.^1^^, ^^2^ The incidence of contrast-induced nephropathy (CIN) in the general population is reported to be 0.6%–2.3%.^3^ The diagnosis of CIN encompasses 3 main components: a sudden increase of 25% or more or an absolute increase of 0.5 mg/dL or more in serum creatinine from the baseline value at 48–72 hours following the exposure to the contrast medium, a temporal relationship between the use of the contrast agent and increased serum creatinine, and the exclusion of other causes for renal insufficiency (e.g., embolism).^4^^-^^7^ The risk of developing CIN is related to the patient, the contrast medium, and the procedure factors. The patient-related risk factors include pre-existing renal failure, cardiovascular problems such as congestive heart failure (New York Heart Association [NYHA] III/IV; NYHA Functional Classification provides a simple way of classifying the extent of heart failure. It places patients in one of four categories based on how much they are limited during physical activity; the limitations/symptoms are in regard to normal breathing and varying degrees in shortness of breath and/or angina.), acute myocardial infarction, hypertension, hypotension, cardiac shock, peripheral vascular disease, age above 75 years, anemia, use of nephrotoxic drugs, periprocedural high serum creatinine (creatinine clearance < 60 mL/min), diabetic nephropathy, and other renal diseases. Some procedures lead to CIN such as the use of an intra-aortic balloon pump, bypass graft intervention, and delayed reperfusion. Also, the high total dose, high osmolality, and high ionic content of the contrast agent are considerable risk factors.^8^^, ^^9^ Several studies have reported an increase in acute renal failure following the administration of the contrast medium in patients undergoing percutaneous coronary intervention (PCI).^10^^, ^^11^ Although it has been shown that the overall incidence of kidney injury after PCI is low without risk factors such as diabetes and pre-existing renal diseases,^12^ CIN is a frequent complication following primary PCI in acute myocardial infarction, even in patients without other risk factors.^13^ In the multivariate analysis of studies on patients undergoing coronary angiography, kidney injury was correlated with high baseline serum creatinine, acute myocardial infarction, shock, older age, insulin-dependent diabetes, and volume of the contrast medium. Kidney injury is associated with increased morbidity, mortality, major cardiovascular events, and prolonged hospitalization.^14^^-^^16^

L-carnitine (beta-hydroxy-gamma-trimethyl amino butyric acid) plays an important role in supporting the body’s metabolic activities.^17^ In the recent years, studies have revealed that L-carnitine has renoprotective effects.^18^ Owing to its antioxidant,^19^^, ^^20^ antiapoptotic,^19^^, ^^21^^, ^^22^ and anti-inflammatory properties, L-carnitine can be considered a preventive treatment against nephrotoxic agents such aminoglycosides, anticancer drugs, and contrast medium agents.^18^

In the present study, we sought to investigate the efficacy of L-carnitine in protecting the kidney from CIN in the catheterization procedure in patients undergoing elective PCI in non-emergency situations.

## Method

This randomized open-labeled clinical trial recruited 202 patients (91 patients in the treatment group and 111 patients in the control group) undergoing PCI in Tehran Heart Center, Tehran University of Medical Sciences, Tehran, Iran, between April 2013 and October 2014. Our study was approved by the institutional Review Board and the Ethics committee of Tehran Heart Center. The patients who were candidated for elective PCI were included. Patients who met at least 1 of the following criteria were excluded from the study: acute coronary syndrome and ST-elevation myocardial infarction, history of PCI or coronary artery bypass graft surgery in the previous 6 months, and impaired renal function (creatinine clearance < 30 mL/min).These conditions may interfere and confound the analysis of kidney damage caused by contrast media.

This study was designed as a randomized open-labeled clinical trial, in which only a nurse knew who received or did not receive L-carnitine. All the patients or their legally authorized representatives gave written informed consent before admittance to the trial and randomization. The permuted-block randomization method with a block size of 4 was used to randomly assign the patients to either the treatment group or the control group. The allocations were concealed until the patients’ consent had been obtained.

The patients were divided into 2 groups: Group 1 (treatment group) received 1 g of L-carnitine (oral vial; So. Se. PHARM; Italy) 3 times a day, 24 hours before the procedure (3 g before PCI) and 2 g after PCI, whereas Group 2 (control group) did not receive L-carnitine. A 5-mL sample of blood was obtained from each patient before receiving the 1st dose of L-carnitine and 12 hours after PCI and was anonymously sent to the local laboratory to be centrifuged. The serum was stored at –70 °C. Target-lesion revascularization was always performed based on angiographic results for all the patients. Coronary angiography and PCI were performed via the femoral artery. The patients received iso-osmolar or low-osmolar and nonionic contrast agent (iodixanol, iopromide, and iohexol). The determination of the dose of the contrast agent was dependent on the PCI procedure. The standard pre-PCI pharmacological therapy included oral aspirin (325 mg) and clopidogrel (300–600 mg). Post-PCI medications included clopidogrel (75 mg), aspirin (80 mg), and atorvastatin (80 mg) daily. Other medications such as beta-blockers and antihypertensive agents were administrated based on the patients’ needs. The patients were discharged 24 hours after PCI.

**Figure 1:**
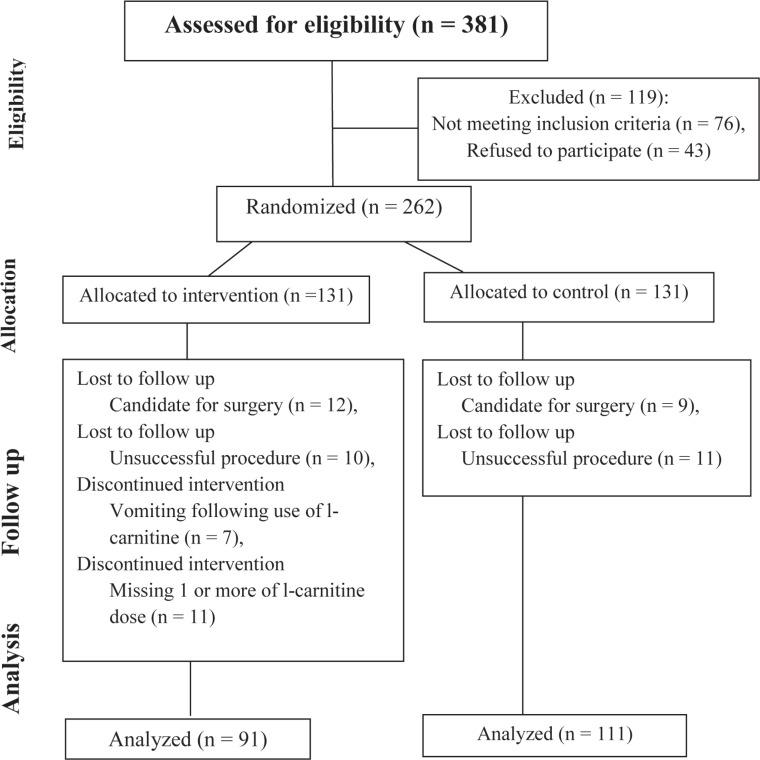
Enrollment of the patients scheduled for elective percutaneous coronary intervention.

The main end point was alteration in plasma neutrophil gelatinase-associated lipocalin (NGAL; NGAL is used as a biomarker of kidney injury. It is expressed in neutrophils and in low levels in the kidney, prostate, and epithelia of the respiratory and alimentary tracts) 12 hours after the PCI and the administration of the contrast medium. The enzyme-linked immunosorbent assay kits were utilized to measure the plasma NGAL (BioVendor, Laboratorní Medicínaa.s., Denmark). NGAL was expressed as its total concentration in plasma (ng/mL).

For the statistical analyses, the statistical software SPSS, version 21 for Windows (IBM, Chicago, Ill), was used. Median (range), mean (SD), and frequency (percentage) were reported to describe the study population. According to the Kolmogorov–Smirnov test of normality, the independent-sample *t*-test or the Mann–Whitney *U*-test was performed to compare the continuous variables. The χ^2 ^test was used to compare the categorical data between the 2 groups. The Spearmen rank test was applied to estimate the correlation between the continuous variables and NGAL. A p value < 0.05 was considered statistically significant.

## Results

The study population was comprised of 202 patients (91 patients in the treatment group and 111 patients in the control group), including 138 (68.3%) males, at a mean age of 60 ± 10.5 years (between 31 and 90 years old) undergoing percutaneous transluminal coronary angioplasty in Tehran Heart Center during an 18-month period. We lost some patients, especially in the treatment group, because of vomiting following the consumption of L-carnitine or missing 1 or more doses of L-carnitine; consequently, we did not have an equal number of patients in the 2 parallel groups (Figure 1). 

The age of the patients was not similar between the 2 randomized groups (Table 1). The other characteristics were the same between the 2 groups such as diabetes mellitus, family history of cardiovascular diseases, serum creatinine, stent diameter, and stent length. Differences in the kind and volume of the contrast medium agent can distort the result, but the administrated volume (p value = 0.313) and the type of the contrast medium (p value = 0.273) were similar between the 2 groups in our study. There was just a single important difference between the case and control groups (age of the patients), which could potentially distort the assessment of the efficacy of L-carnitine. Therefore, we adjusted the result of the study statistically based on the confounding factors.

A higher baseline plasma level of NGAL was significantly related to having a family history of cardiac diseases (p value < 0.001), lower BMI (p value = 0.008), and higher troponin T level (p value = 0.011); these differences, however, failed to constitute statistical significance.

**Figure 2:**
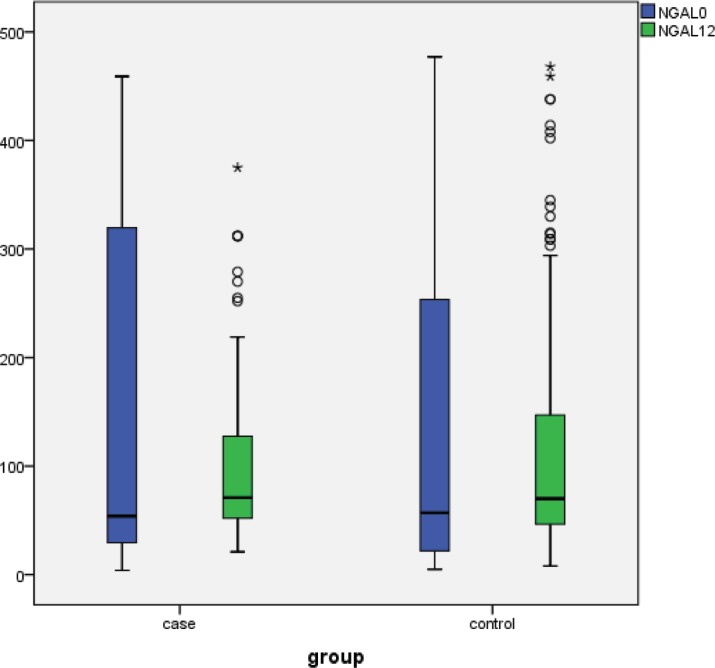
Minimum, maximum, and percentile of neutrophil gelatinase-associated lipocalin (NGAL) concentrations at baseline and 12 hours post intervention according to the study groups

Totally, NGAL-12 was correlated to NGAL-0 (r = 0.715; p value < 0.001). The baseline median plasma level of NGAL (NGAL-0) was not different between the treatment and control groups (p value = 0.155). Also 12 hours after PCI, the plasma level of NGAL was not different between the 2 groups (p value = 0.925). Nonetheless, the changes in NGAL were different between the groups, and there was an interaction between time and group. NGAL increased in the control group (from baseline to 12 hours after PCI) but nonsignificantly (p value = 0.051), whereas in the L-carnitine group, the changes were not significant (p value = 0.425). In other words, the changes were higher in the control group than in the L-carnitine group (median of differences = 17 ng/mL [IQR = -21–41] vs. 5 ng/mL [IQR = -147–30]; p value = 0.010) (Table 2; Figure 2).

**Table 1:** Patients’ demographic and clinical characteristics based on the study groups*

Factors	Total	Carnitine Group	Control Group	P value
Number of Patients	202	91 (45.1)	111 (54.9)	
Age (y)	60.0±10.5	62.0±9.0	57.0±11.2	< 0.001
Female Sex	64 (31.7)	31 (34.1)	33 (29.7)	0.510
BMI (kg/m^2^)	27.0±4.3	27.1±4.2	26.9±4.3	0.746
Family History for CAD	72 (35.6)	30 (33.7)	42 (37.8)	0.472
Current Smoker	76 (37.6)	30 (33.7)	46 (41.4)	0.216
Dyslipoproteinemia	103 (50.9)	40 (43.9)	59 (53.1)	0.497
Hypertension	122 (60.4)	51 (56.1)	71 (63.9)	0.252
Diabetes Mellitus	52 (25.7)	20 (22.0)	32 (28.8)	0.268
Opium	17 (8.4)	6 (6.6)	11 (9.9)	0.398
Ejection fraction	50 (15-70)	50 (15-65)	50 (35-70)	0.442
Medication History				
Statin	137 (67.8)	61 (67.0)	76 (68.5)	0.828
Beta-blocker	140 (69.3)	58 (63.7)	82 (73.9)	0.120
ACEI	69 (34.2)	28 (30.8)	41 (36.9)	0.358
ARB	76 (37.6)	39 (42.8)	37 (33.3)	0.164
Clopidogrel	85 (42.1)	42 (46.2)	43 (38.7)	0.288
Aspirin	178 (88.1)	82 (90.1)	96 (86.5)	0.428
Antidiuretic agent	61(30.2)	25 (27.5)	36 (32.4)	0.455
Anti-ischemic	145 (71.8)	71 (78.0)	75 (67.6)	0.099
Diuretics	88 (43.5)	39 (42.8)	49 (44.1)	0.203
Serum creatinine	0.86 (0.46-1.60)	0.85 (0.56-1.40)	0.86 (0.46-1.60)	0.399
Troponin T	11.86 (3-3057)	12.39 (3-3057)	11.18 (3-2013)	0.210
CK-MB	1.70 (0.48-11.18)	1.60 (0.86-9.99)	1.75 (0.48-11.18)	0.255
Angiography Result				0.525
SVD	80 (39.6)	37 (40.6)	43 (38.7)	
2VD	77 (38.1)	37 (40.6)	40 (36.0)	
3VD	45 (22.2)	17 (18.7)	28 (25.2)	
Type of stents				0.343
DES	180 (89.1)	79 (86.8)	101 (91.9)	
BMS	22 (10.9)	12 (13.2)	10 (9.0)	
Stent No.				0.342
1	170 (84.2)	79 (86.8)	91 (81.9)	
2	32 (15.8)	12 (13.2)	20 (18.0)	
Stent Length (mm)	28 (12-82)	28 (12-68)	30 (12-82)	0.108
Contrast type				0.273
Iodixanol (320)* *; Osmolality (290) ***	147 (72.8)	72 (79.1)	75 (67.6)	
Iopromide (300) **; Osmolality (607) ***	15 (7.4)	4 (4.4)	11 (9.9)	
Iopromide (370) **; Osmolality (774) ***	27 (13.4)	10 (10.9)	17 (15.3)	
Iohexol (350) **; Osmolality (844) ***	13 (6.4)	5 (5.5)	8 (7.2)	
Contrast volume (mL)	200 (100-500)	200 (100-500)	200 (100-500)	0.313

BMI, Body mass index; CAD, Coronary artery disease; ACEI, Angiotensin-converting enzyme inhibitor; ARB, Angiotensin-receptor blocker; CK-MB, Creatine kinase–MB; SVD, Single-vessel disease; 2VD, 2-vessel disease; 3VD, 3-vessel disease; DES, Drug-eluting stent; BMS, Bare-metal stent

* Data are presented as means±SD, medians (interquartile range), or n (%)

** Generic name of the contrast medium agent (mgI/mL)

*** Osmolality (mosm/kg of water)

**Table 2:** Level of NGAL at baseline and after 12 hours according to the study groups*

Factor	Total	L-Carnitine Group	Placebo Group	P value**
Baseline	55.5 (25.5-267)	54 (29-324)	57 (22-255)	0.155
Post 12 h	71 (48-133.5)	71 (52-129)	70 (46-153)	0.925
Difference 0–12 h	11.5 (-43.25-34)	5 (-147 - 30)	17 (-21-41)	0.010
P value**	0.052	0.425	0.051	

NGAL, Neutrophil gelatinase-associated lipocalin

* Data are presented as median (interquartile range).

** P value reported for subgroup comparison differences

## Discussion

The most important finding of the present study was the demonstration of a significant alteration in the plasma NGAL level by L-carnitine administration in contrast-medium-exposed patients undergoing PCI. 

The most frequently reported hypothesis for the mechanism of CIN is based on decreased renal blood flow and oxygen delivery secondary to renal artery vasoconstriction by the action of adenosine and endothelin and also the impairment of the action or production of vasodilators such as nitric oxide and prostaglandin. Also, the contrast medium has direct toxic effects on proximal tubules due to vacuolization, changes in mitochondrial function, apoptosis, and necrosis.^14^^, ^^15^ Many experimental studies have shown that L-carnitine reduces drug-induced nephropathy via several mechanisms such as anti-inflammatory effects, antioxidative properties by the inhibition of reactive oxygen species (ROS) generation and lipid peroxidation, inhibition of matrix remodeling and apoptosis, and also improvement in carnitine deficiency.^18^ Therefore, antioxidant agents have been considered against CIN due to their antioxidant properties.^23^ There are no human clinical studies on the protective effects of L-carnitine against CIN. A review of literature from Jafari et al.^18^ summarized the nephroprotective effects of L-carnitine against drug-induced nephropathy. The authors reviewed the effects of L-carnitine against nephropathy following the administration of medications such as platinum derivatives, oxazaphosphorines, doxorubicin, methotrexate, calcineurin inhibitors, and contrast agents and reported that L-carnitine significantly reduced drug-induced nephropathy in animal studies, especially against cisplatin-induced renal damage. The investigators also reported that other information was very limited to support the efficacy of L-carnitine and suggested that further well-designed human studies were needed.

We herein summarize a number of animal studies that have examined the protective effects of L-carnitine. Jabbari et al.^24^ in their study evaluated the high and low doses of L-carnitine against CIN in rats. The authors showed the nephroprotective impact of L-carnitine, particularly at a high dosage of 200 mg/kg/d. They reported that L-carnitine decreased the severity of contrast-induced proximal tubular necrosis and renal dysfunction. In their study, the diatrizoate meglumine + high dose L-carnitine group had less severe proximal tubular necrosis than the rats in the meglumine diatrizoate + low dose L-carnitine or meglumine diatrizoate only groups. Kunaka CS et al.^25^ examined the nephroprotective effects of carnitine against glycerol and contrast-induced kidney injury in rats. They showed that L-carnitine significantly enhanced antioxidant levels, decreased oxidative parameters such as pro-inflammatory cytokines and apoptosis biomarkers, and reduced elevated serum creatinine and blood urea nitrogen in contrast medium-induced nephrotoxicity in rats with underlying pathology. 

Although the protective effects of many compositions and medications for the prevention of CIN have been investigated, preventive measures are accepted by all clinicians.^26^^, ^^27^ The incidence or severity of CIN can be reduced by appropriate risk stratification, sufficient hydration with normal saline or sodium bicarbonate, withholding of nephrotoxic medications, use of low- or iso-osmolar contrast media, or contrast dose reduction. N-acetylcysteine administration is popular in clinical settings because of its high efficiency and minimal side effects. Most trials have indicated that N-acetylcysteine, particularly when associated with adequate hydration, might be useful in preventing CIN.^18^^, ^^28^^-^^30^

The overall incidence of kidney injury after PCI, accompanied by the administration of the contrast medium, is low; nonetheless, diabetic patients and especially those suffering from chronic kidney diseases with a serum creatinine level above 2.0 are at high risk for acute kidney injury (AKI) after PCI.^31^ Fortunately, we did not find significant differences in the demographic and clinical characteristics between our 2 study groups except the age of the patients. Older age may increase the risk of renal damage in patients undergoing PCI, but the strength of this relation is weak. Although we adjusted the results based on confounding factors by statistical methods, the same results were obtained. 

In the present study, the patients were discharged 24 hours after PCI. Therefore, serum creatinine as the gold standard biomarker for CIN diagnosis was not measurable 72 hours after PCI. The serum creatinine level has a slow rate of plasma change and can delay the early diagnosis of kidney injury. On the other hand, recent studies have confirmed the value of new biomarkers for the detection of kidney damage. Until now, the serum creatinine level and urine output have been the most frequently used indicators of renal function, thus limiting their usefulness in the early detection of AKI.^32^ They have limited sensitivity and specificity for the detection of renal dysfunction. Early detection of AKI by novel biomarkers can contribute to proper medical proceedings, which combined with the monitoring of response to therapy, could prevent more renal damage. Some of these biomarkers that reflect kidney damage include kidney injury molecule-1 (KIM-1), NGAL, and interleukin-18. Patients with subclinical AKI may be recognized as soon as possible by following renal damage markers.^19^ In the recent years, several studies have assessed the clinical significance of the application of new biomarkers for the detection of early kidney damage. Two significant issues vis-à-vis kidney damage should be borne in mind: kidney damage alone and functional change following the damage. The biomarkers that reflect kidney damage (e.g., KIM-1, NGAL, and interleukin-18) can be utilized for the early detection of kidney damage even without functional damage. The development of validated early biomarkers to detect drug-induced nephropathy may give us a golden opportunity to use prophylactic nephroprotective agents at the right time. NGAL is an early biomarker for a structural renal injury and reflects damage to the kidney with or without functional change. NGAL is highly sensitive; thus, it is a good candidate for the detection of early minor or major kidney damage. However, the measurement of new biomarkers like NGAL after PCI is allied to several limitations such as varying results and different values for the prediction of contrast-induced AKI.^33^^-^^37^ However, different values of NGAL for AKI prediction have been reported in clinical studies. By using standardized clinical platforms, an NGAL cutoff value more than 150 ng/mL in studies has been identified as AKI, while higher or lower cutoff values have been used in different clinical studies. To find out what constitutes the true cutoff value, we need further studies enrolling patients with normal or impaired renal function in both patients developing AKI and those without AKI.^38^ Although there are limited data on NGAL measurements after PCI, the predictive role of NGAL in the early diagnosis of CIN has already been shown in adults and children undergoing coronary angiography.^37^^, ^^39^^, ^^40^

The decision was difficult for us to choose the suitable doses of L-carnitine. The administration of higher doses of L-carnitine more than 3 g per day can cause nausea and stomachache, and we had to divide the total dose of L-carnitine. The total dose of 5 g is based on limited data from a few previous human studies on drug-induced nephrotoxicity (not just CIN).^18^

In our study, 36 patients in the control group and 33 patients in the treatment group had baseline serum NGAL levels greater than 150 ng/mL, whereas none of the patients had renal insufficiency because serum creatinine was normal in all the included patients and we excluded high-risk patients for renal injury (except diabetic patients with a normal renal function). Based on the obtained results, both of our study groups had a rise in their NGAL level; nevertheless, in the L-carnitine group, the change was not significant. This finding shows that L-carnitine canto some extent prevent NGAL from rising in patients who receive the contrast medium. The present study is one of the 1st human trials to show the protective effects of L-carnitine against CIN. 

The present study suffers from some limitations, first and foremost among which is that it was difficult for us to choose the suitable doses of L-carnitine and its administration schedule. Furthermore, the sample volume in our study was insufficient, and we had unequal numbers of patients in the 2 groups. Another drawback of note is the absence of a long-term follow-up of the patients. We recommend that the other biomarkers and diagnostic factors of kidney damage and function be taken into account in future studies with a view to clearly delineate the clinical benefits of the efficacy of L-carnitine against CIN. 

## Conclusion

Although we showed that oral L-carnitine was able to prevent an increase in NGAL or decrease NGAL following the administration of the contrast medium in patients undergoing PCI, more studies are needed to clarify the clinically-proven nephroprotective effects of L-carnitine against CIN. 

## Notes:

***This paper should be cited as:*** Mohammadi M, Talasaz AH, Alidoosti M, Pour Hosseini HR, Gholami K, Jalali A, Aryannejad H. Nephroprotective Effects of L-Carnitine against Contrast-Induced Nephropathy in Patients Undergoing Percutaneous Coronary Intervention: A Randomized Open-Labeled Clinical Trial. J Teh Univ Heart Ctr 2017;12(2)57-64.
